# The Evaluation Point-of-Care Ultrasound in the Post-Anesthesia Unit–A Multicenter Prospective Observational Study

**DOI:** 10.3390/jcm10112389

**Published:** 2021-05-28

**Authors:** Davinder Ramsingh, Sumit Singh, Cecilia Canales, Elyse Guran, Zach Taylor, Zarah Antongiorgi, Maxime Cannesson, Robert Martin

**Affiliations:** 1 Department of Anesthesiology, Loma Linda University Medical Center, Loma Linda, CA 92354, USA; EGuran@llu.edu (E.G.); ZTaylor@llu.edu (Z.T.); RDMartin@llu.edu (R.M.); 2 Department of Anesthesiology & Perioperative Medicine, David Geffen School of Medicine at UCLA, Los Angeles, CA 90095, USA; SPSingh@mednet.ucla.edu (S.S.); CeciliaCanales@mednet.ucla.edu (C.C.); ZAntongiorgi@mednet.ucla.edu (Z.A.); MCannesson@mednet.ucla.edu (M.C.)

**Keywords:** point-of-care ultrasound, post-anesthesia care unit, hemodynamics, hypoxia, hypotension, anesthesia

## Abstract

Introduction: Point-of-care ultrasound (POCUS) is the most rapidly growing imaging modality for acute care. Despite increased use, there is still wide variability and less evidence regarding its clinical utility for the perioperative setting compared to other acute care settings. This study sought to demonstrate the impact of POCUS examinations for acute hypoxia and hypotension occurring in the post-anesthesia care unit (PACU) versus traditional bedside examinations. Methods: This study was designed as a multi-center prospective observational study. Adult patients who experienced a reduced mean arterial blood pressure (MAP < 60mmHG) and/or a reduced oxygen saturation (SpO2 < 88%) in the PACU from 7AM to 4PM were targeted. POCUS was available or not for patient assessment based on PACU team training. All providers who performed POCUS exams received standardized training on cardiac and pulmonary POCUS. All POCUS exam findings were recorded on a standardized form and the number of suspected mechanisms to trigger the acute event were captured before and after the POCUS exam. PACU length of stay (minutes) across groups was the primary outcome_._ Results: In total, 128 patients were included in the study, with 92 patients receiving a POCUS exam. Comparison of PACU time between the POCUS group (median = 96.5 min) and no-POCUS groups (median = 120.5 min) demonstrated a reduction for the POCUS group, *p* = 0.019. Hospital length of stay and 30-day hospital readmission did not show a significant difference between groups. Finally, there was a reduction in the number of suspected diagnoses from before to after the POCUS examination for both pulmonary and cardiac exams, *p*-values < 0.001. Conclusions: Implementation of POCUS for assessment of acute hypotension and hypoxia in the PACU setting is associated with a reduced PACU length of stay and a reduction in suspected number of diagnoses.

## 1. Introduction

Recently, the application of point-of-care ultrasound has gained significant interest from anesthesiologists. A simple keyword search for “perioperative point-of-care ultrasound” in PubMed results in just over 200 publications on the topic, with 95% being published in the past 10 years and 78% being published in the past 5 years (accessed on 24 April 2020). Point-of-care ultrasound (POCUS) can be defined as the use of ultrasonography at the patient’s bedside for diagnostic and therapeutic purposes [1]. Fundamental to POCUS is the practice in which the sonographer acquires and interprets the exam in real time and subsequently uses this information to diagnose and direct therapies. This unique imaging modality can provide insight into acute events and has the added benefit of being simple, rapid, and goal-orientated, with the option for more comprehensive point-of-care imaging, if needed [2].

In the perioperative setting, POCUS has demonstrated utility for examining nearly all components of bedside assessment, with the majority of evidence for cardiovascular and pulmonary evaluation [2,3]. Application of cardiovascular and pulmonary POCUS examination by anesthesiologists has been shown to accurately detect significant pathologies and impact perioperative management decisions [3,4,5]. With growing research demonstrating the efficacy of perioperative POCUS, more anesthesiologists are incorporating POCUS into their clinical management skillsets. A recent study demonstrated the impact of a perioperative POCUS service, which resulted in improved diagnostic accuracy of new pathologies, as well as severity assessment of known pathologies when compared to traditional assessment techniques [3].

Additionally, multiple cardiopulmonary protocols have been reported to be useful for the perioperative setting [5,6,7,8,9]. This was discussed in a recent consensus paper reporting a “call to action” on this topic [10], as well as in a recent clinical focus review article [2]. In the United States, further support for implementing perioperative POCUS curricula was established by the Accreditation Council for Graduate Medical Education (ACGME) with updated 2018 program requirements, listing “competency in using surface cardiac and pulmonary ultrasound to evaluate organ function and pathology.” [11].

While the growth in research on the clinical utility and effectiveness of educational programs has helped validate the importance of POCUS application to the perioperative setting, its direct impact on performance markers of perioperative patient care has been lacking. PACU length of stay (LOS) is of key importance to anesthesiologists as it can often impact operating room (OR) workflow and case load [12]. Episodes of hypoxia and hypotension are common postoperative complications in the PACU setting [13,14]. Since the perioperative POCUS has been shown to improve the diagnostic accuracy of acute pathology, this study sought to evaluate the impact of applying a validated POCUS protocol in the PACU for patients with hypoxic and/or hypotensive events. To strengthen transferability, this study was performed simultaneously at two major academic centers. We hypothesized that the application of POCUS for these specific acute events would demonstrate a reduction in PACU LOS compared to patients who had these events but did not receive a POCUS examination.

## 2. Methods

Institutional Review Board approval was obtained at both institutions (IRB # 5170140- LLU and IRB # 17-000730-UCLA) as a prospective, observational study (clinicaltrials.gov ID: NCT04410757). The study was performed in the respective PACU covering the main medical center for both tertiary care academic hospitals. The study was implemented to assess current practice environments in which some attending anesthesiologists supervising PACU patient care are POCUS trained while others are not. POCUS training was standardized across both institutions using a validated online curriculum [3] and was voluntary. Data was collected from August 2018 to August 2019. Given this study design, it is reported following the Standards for Quality Improvement Reporting Excellence (SQUIRE) guidelines.

## 3. Development of the Training Curriculum

Prior to initiation of the study an educational curriculum was developed to train attending anesthesiologists at both institutions. This curriculum was based on selected cardiopulmonary topics from a previously validated online perioperative ultrasound educational course abbreviated FORESIGHT (Focused periOperative Risk Evaluation Sonography Involving Gastro-abdominal, Hemodynamic, and Trans-thoracic ultrasound) [3]. Specific topics from the FORESIGHT curriculum included transthoracic echocardiography, pneumothorax assessment, pleural effusion assessment, pulmonary parenchyma disease, and detection of endotracheal tube (ETT) malposition. Completion of training was determined by verification of online content review and supervised performance of at least 50 exams, as previously supported [5].

## 4. Designing the Intervention

Adult patients were enrolled in the study during the hours of 7 AM to 4 PM to evaluate impact during routine clinical hours. The availability of POCUS was determined at the start of the clinical day (7 AM) based on whether the attending anesthesiologist assigned to cover the PACU was trained on POCUS or not. Study inclusion criteria consisted of admission to PACU, > 18 years of age, documented event of mean arterial blood pressure <60 mmHG, and/or pulse oximetry saturation of <88%. Exclusion criteria included emergent procedures and a failure to improve vital signs prior to transfer out of the PACU.

## 5. Process Measures

A waiver of consent was approved by both academic centers given the status of the patient that was being enrolled and the observational study design. Examinations were documented on the same form at both institutions. (Appendix A) The EMR systems (Epic systems) were accessed to review for PACU LOS times retrospectively. A data share agreement was developed between both institutions prior to study launch.

## 6. Outcome Measures

The primary outcome marker for the study was PACU LOS (minutes), with a hypothesis that patients receiving a POCUS examination for the cardiovascular or pulmonary event would have a shorter PACU LOS. PACU LOS was defined as the time from admission to the PACU to the time when the patient was deemed “ready for discharge” by the PACU provider, identified as the time when the PACU discharge note was placed in the EMR. The Aldrete Discharge Score, which evaluates respiration, circulation, consciousness, color, and mobility, was used at both institutions to determine whether patients were ready for discharge. A score of >8 out 10 was required for PACU discharge approval. When a POCUS exam was performed, a report (Appendix A) was completed by the performing attending anesthesiologist that included pertinent findings in addition to the number of potential diagnoses to explain the acute event (hypoxia and/or hypotension) before and after the POCUS exam.

## 7. Devices Used

Multiple ultrasound systems were used during the study, including laptop-based systems (Fujifilm Sonosite Bonethell, WA, USA, General Electric, Boston, MA, USA) and handheld devices (Butterfly Network Guilford, CT, USA).

## 8. Statistical Analysis

This study planned to evaluate continuous non-paired parametric data via the two-sample t-test and paired continuous variables via the paired *t*-test. All continuous variables failing to meet parametric assumptions, were analyzed using a Mann–Whitney test for non-paired data and the Wilcox test for paired data. Categorical variables were analyzed using a Chi-Squared test when assumptions were met, otherwise the Fishers exact test was used. The Shapiro–Wilk test was used to evaluate normality for continuous primary and secondary outcomes. Moreover, the Levene’s test was used to evaluate variance assumptions of continuous variables. The primary outcome was a comparison in the differences in central tendency of PACU time across the POCUS and no-POCUS groups. Secondary outcomes included comparisons across POCUS and no-POCUS groups for 30-day hospital readmission, and hospital length of stay. Additionally, the number of possible diagnoses that triggered the hypotension and/or hypoxic event pre-POCUS was compared to the number post-POCUS assessment for both pulmonary and cardiac examinations. All analyses were conducted in R version 3.5.0 R Core Team. (2020). (R: A Language and Environment for Statistical Computing. Vienna, Austria. Retrieved from https://www.R-project.org/, accessed on 20 May 2021).

## 9. Sample Size

Sample size was determined for the primary outcome marker of a reduction in PACU time between the POCUS and no-POCUS groups. A significant reduction in PACU time was established, a priori, at 15%. Given the level of POCUS training and the observational study design, enrollment was based on two-thirds of the patients receiving POCUS and one-third of the patients not. The sample size for this study was calculated for a two-sample t-test where PACU time, a continuous variable, was the primary outcome of comparison between the POCUS and no-POCUS groups. Assuming an alpha = 0.05, power = 0.80, the allocation ratio (POCUS/PE) of 3, and an effect size of 0.6, we identified a sample size of 90 patients in POCUS group and 30 patients in the no-POCUS group.

## 10. Results

A total of 128 patients were included in the study with 92 patients receiving a POCUS exam and 36 patients not receiving a POCUS exam during evaluation of the acute event. A total of 7 clinicians performed the POCUS exams. Median and interquartile range (IQR) for baseline demographics are reported in Table 1.

**Figure 1:**
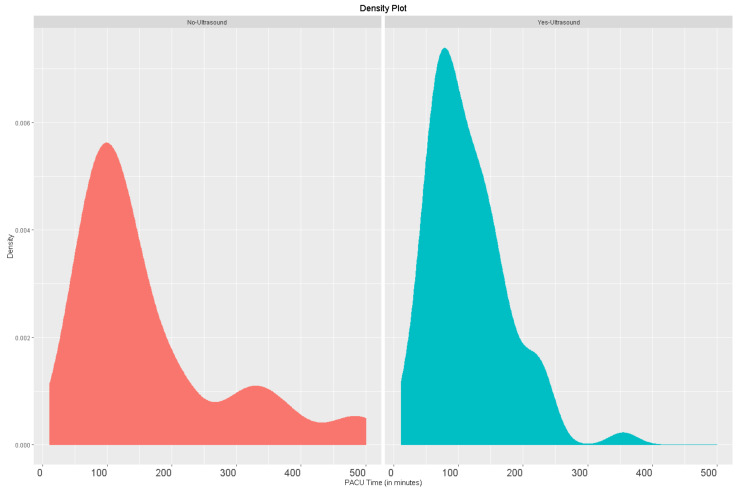
Comparison of post-anesthesia care unit length of stay (minutes).

The Shapiro–Wilk test showed lack of normality (*p*-value < 0.01) for all numeric primary and secondary outcomes. Comparison of PACU LOS between the POCUS group (median/IQR = 96.5/77.5 min) and no-POCUS group (median IQR = 120.5/121.5 min minutes) demonstrated a significant reduction for the POCUS group, *p* = 0.019 (Table 2/Figure 1).

Hospital length of stay did not show a significant difference between groups, *p*-values = 0.094. Moreover, there was no difference in 30-day hospital readmission across POCUS and no-POCUS groups (*p* = 1). Finally, there was a significant reduction in the number of suspected diagnoses from before to after POCUS for both pulmonary and cardiac exams, with *p*-values of <0.001 and <0.001, respectively (Table 3). The most common mechanisms identified by the cardiac POCUS exam were hypovolemia (50%), distributive shock (21%), and depressed ejection fraction (11%). The most common mechanisms identified by the pulmonary POCUS exams were airspace disease/atelectasis (41%), pulmonary edema (32%), and pleural effusion (18%).

## 11. Discussion

The evidence for the utility of POCUS in the perioperative setting has dramatically increased over recent years. However, most of these studies have reported on the accuracy of anesthesiologists’ performed POCUS examinations as well its impact on clinical management decisions. While these data encourage POCUS integration into perioperative care, further support can be gained through demonstration of POCUS application directly improving valued perioperative care metrics. This study validates the utility of POCUS for patients who have a hypotensive and/or hypoxic event in the PACU by demonstrating a shortened PACU LOS when POCUS was utilized. Supporting this, our findings indicate a statistically significant reduction in the number of suspected mechanisms for the acute event after POCUS was performed. These results suggest that attending anesthesiologists were able to reduce the PACU LOS by determining the likely mechanism more definitively with POCUS application and thus provide therapy in a timelier manner.

The importance of a PACU service is essential for the early detection of postoperative complications [15]. Practice guidelines from the American Society of Anesthesiologists state that patients should not be discharged from the PACU setting while still at increased risk for cardiorespiratory depression [16]. Nevertheless, anesthesiologists are pressured to decrease PACU length of stay and studies have shown a cost savings associated with reduced PACU time [17]. This study was supported across both institutions with the goal of understanding and ultimately reducing PACU time to improve perioperative workflow. Lack of PACU bed availability causes retention of cases in the OR, which delays subsequent surgeries and in extreme examples utilizes OR staff to recover patients intraoperatively. Incorporation of clinical pathways have been effective towards reducing PACU time, with the positive impact secondary to early recognition and management of post-operative complications [18]. The results of our study suggest that POCUS may also allow for improved throughput via the same mechanism, which ultimately may lead to a cost savings if applied consistently.

Indeed, the ability of POCUS to decrease LOS has been demonstrated in other areas outside of the perioperative setting. Multiple studies have shown that POCUS application can decrease LOS in the emergency department [19,20,21], in the inpatient internal medicine setting [22], as well as intensive care units [23]. To the authors’ knowledge, this study is the first to show an association between POCUS and a reduced LOS in the PACU setting.

This study has several limitations to consider, including the prospective observational design. Given the difficulty of predicting which patients would experience hypotension or hypoxia in the PACU setting, we were unable to identify a strategy that would allow us to consent patients to participate in a randomized controlled trial comparing POCUS to no-POCUS PACU management strategies. Additionally, this study design evaluated all patients recovering in the PACU and our analysis was not powered to include confounding variables, such as patient demographics, comorbidities, surgical procedure, intraoperative blood loss, fluid administration, and vasoactive medications. These are important limitations to this novel study. Additionally, while both institutions followed criteria to determine readiness for PACU discharge, variability in clinicians’ assessment must be recognized. Moreover, the ability to provide POCUS training to all anesthesiologists who supervise the PACU environment at both institutions was not feasible. We sought to address these limitations by performing this study across two different academic medical centers and include examinations only performed during standard working hours (7 AM to 4 PM). Regarding POCUS competency, all attending faculty instructors were required to have completed the online training and 50 FORESIGHT examinations as previously validated [3]; however, there is no current standard perioperative POCUS certification pathway.

## 12. Conclusions

This project demonstrates that the novel application of POCUS in the PACU setting for patients who are hypotensive or hypoxic is associated with a shortened PACU LOS and results in a reduction in suggested mechanisms contributing to the acute event. Additional studies should evaluate how POCUS implementation can provide improvement in other perioperative outcomes.

## Figures and Tables

**Table 1:** Demographic and surgery type data.

Group	Count	POCUS	No POCUS
		*n* = 92	*n* = 36
Age (in years)	Median (IQR)	65.00 (23.75)	65.00 (28.50)
Sex			
Male:Female	Count (Percentage)	53 (58%):39 (42%)	18 (50%):18 (50%)
Weight (in kg)	Median (IQR)	77.50 (29.92)	71.80 (17.37)
Height (in cm)	Median (IQR)	154.00 (168.82)	161.15 (170.28)
BMI	Median (IQR)	26.70 (7.84)	24.97 (5.68)
ASA			
1		1(1%)	0(0%)
2		12(13%)	8(22%)
3	Count (Percentage)	58(64%)	21(59%)
4		21(22%)	7(19%)
Surgery Type			
Head and Neck		6 (7%)	4 (11%)
Thorax Surgery		16 (17%)	4 (11%)
Abdominal		15 (16%)	6 (17%)
Urologic		4 (4%)	2 (6%)
Gynecologic/Obstetric		5 (5%)	2 (6%)
Orthopedic		9 (10%)	8 (22%)
Vascular		20 (22%)	6 (16%)
Other		17 (18%)	4 (11%)

ASA= American Society of Anesthesiology Physical Status Classification, BMI = Body Mass Index.

**Table 2:** Post-surgery outcome comparisons between hypoxic/hypotensive patients; group indicator for subjects with point-of-care ultrasound or without.

		POCUS *n* = 92	No POCUS *n* = 36	95 % CI Estimate (Lower, Upper)	*p*-Values
PACU Length of Stay (in minutes)	Median (IQR)	96.50 (77.50)	120.50 (121.25)	−26.00 (−52.00, −4.00)	0.01877
Hospital Length of Stay (in days)	Median (IQR)	2.50 (5.50)	5.00 (5.00)	−1.00 (−3.00, 0.00)	0.09363

PACU = Post-Anesthesia Care Unit, HLOS = Hospital Length of Stay, POCUS = Point-of-Care Ultrasound.

**Table 3:** Comparisons of the number of suspected mechanisms to trigger PACU cardiopulmonary events.

		Cardiac Pre-POCUS	Cardiac Post-POCUS	95 % CI Estimate (Lower, Upper)	*p*-Values
# of DX	Median (IQR)	2 (2)	1 (0)	1.5 (1.4, 2.0)	<0.001
		**Pulmonary Pre-POCUS**	**Pulmonary Post-POCUS**	**95 % CI** **Estimate (Lower, Upper)**	***p*-Values**
# of DX	Median (IQR)	5 (2)	1 (1)	3.0 (2.5, 4.0)	<0.001

DX = Diagnoses, POCUS = Point-of-Care Ultrasound, PACU = Post-Anesthesia Care Unit, # = Number.

## Data Availability

The data presented in this study are available on request from the corresponding author. The data are not publicly available due to privacy protection.

## Appendix A. PACU POCUS FORM

**Date/Time**: ___________ **Examiner name**: _________________**Attending:** ______________

**Chief Compliant:** ____________________________________________________________________________

**Reason for consult:**

☐ Hypoxia ☐Hypotension

**Relevant Physical Exam Findings:**

**Cardiovascular:** ________________________________________________________________________________

**Pulmonary:** _____________________________________________________________________________________

**Did you use point of care ultrasound to evaluate this patient?** ☐Yes ☐No

**Relevant POCUS findings:_**

**Cardiovascular:** ________________________________________________________________________________

**Pulmonary:**_____________________________________________________________________________________

**Cardiac Diagnosis:**

☐**= WNL**☐Hyperdynamic

☐Systolic Dysfunction ☐Diastolic Dysfunction ☐W.M.A. ☐Arrhythmia ☐Myocarditis

☐Hypertrophic cardiomyopathy ☐Tamponade ☐Pericardial effusion ☐ RV dysfunction ☐ LVH ☐ RVH

☐ R/L Enlarged Atrium ☐ R/L Enlarged Ventricle ☐Pulm HTN

Valvular abnormality: ☐AR ☐AS ☐MR ☐MS ☐PR ☐PS ☐TR ☐TS

**IVC:**

☐**=WNL**☐ <50% collapsibility ☐>50% collapsibility

**Pulmonary Diagnosis:**

☐ **=WNL**

☐Pneumothorax ☐Pulmonary Edema (bilateral B lines) ☐Atelectasis

☐Pleural Effusion

☐Aspiration ☐Pneumonia ☐ET tube placement/location ☐Air Space Disease

**Airway:**☐**=WNL**☐Trachea Compression ☐ Vocal Cord Paralysis ☐ Neck Mass ☐ Mainstem intubation

**Suspected Diagnoses BEFORE Physical Exam or POCUS Exam:**	
**Shock**	**Hypoxia**
☐Distributive shock (sepsis)	☐Pneumonia/consolidation
☐Depressed EF	☐ET tube placement/location
☐Pulmonary Embolus	☐Pleural effusion
☐Pericardial Effusion/Tamponade	☐Pulmonary Edema
☐Hypovolemia/hemorrhage	☐Pneumothorax ☐Atelectasis / Airspace disease
☐Other_________________	☐Other_________________
**Suspected Diagnoses AFTER Physical Exam or POCUS Exam:**	
**Shock**	**Hypoxia**
☐Distributive shock (sepsis)	☐Pneumonia/consolidation
☐Depressed EF	☐ET tube placement/location
☐ Pulmonary Embolus	☐Pleural effusion
☐Pericardial Effusion/Tamponade	☐Pulmonary Edema
☐Hypovolemia/hemorrhage	☐Pneumothorax ☐Atelectasis / Airspace disease
☐Other_________________	☐Other_________________
